# Global transcriptional modulation and nutritional status of soybean plants following foliar application of zinc borate as a suspension concentrate fertilizer

**DOI:** 10.1038/s41598-025-87771-5

**Published:** 2025-01-26

**Authors:** Eloisa Vendemiatti, Rafael Oliveira Moreira, Gabriel Lasmar dos Reis, Inty Omar Hernandez-De Lira, Eugenia Peña-Yewtukhiw, Franz Walter Rieger Hippler, Luis Omar Torres-Dorante, Kiran Pavuluri, Alex Valentine, Vitor L. Nascimento, Vagner Augusto Benedito

**Affiliations:** 1 School of Agriculture and Food Systems, Davis College of Agriculture and Natural Resources, West Virginia University, Morgantown, WV USA; 2 Yara Agronomy and R&D - Research Centre Hanninghof, Yara International S.A, Dülmen, Germany; 3 International Fertilizer Development Center, Muscle Shoals, AL USA; 4 Yara Agronomy and R&D, Yara International S.A, Pocklington, York UK; 5 Department of Biology, Institute of Natural Sciences, Universidade Federal de Lavras, Lavras, MG Brazil; 6 Department of Biology, West Virginia State University, Institute, WV, United States

**Keywords:** Cell wall biosynthesis, Gene expression, Foliar fertilizer uptake, Membrane transport, Micronutrient deficiency markers, Micronutrient homeostasis, Mineral nutrition, Plant physiology, Transcriptomics

## Abstract

The management of micronutrients, such as boron (B) and zinc (Zn), is critical for plant growth and crop yields. One method of rapid intervention crop management to mitigate nutritional deficiency is the foliar supply of B and Zn. Our study investigates the effect of foliar-supplied B and Zn availability on the global transcriptional modulation in soybean (*Glycine max*). The transcriptional response to B was more widespread compared to Zn. RNA-Seq of leaves under different B levels revealed modulated genes with potential roles in nutritional homeostasis and stress response that may be key to controlling B status in the plant. We also identified putative B transporters whose expression levels were significantly affected by B supplementation with foliar fertilization of plants growing under low B conditions. Furthermore, a gene lacking functional annotation (Glyma.03G180300) emerged as a novel potential marker of B status. Two genes (Glyma.16G118000, Glyma.16G199000) were consistently induced by Zn availability, highlighting their potential as biomarkers for assessing its status in soybean plants. This work advances our understanding of nutritional homeostasis in soybean plants and identifies target genes and potential molecular mechanisms involved in nutritional response. Our study informs fertilizer design targeting specific nutrient transporters, thereby enhancing nutrient efficiency in crops.

**Supplementary Information:**

The online version contains supplementary material available at 10.1038/s41598-025-87771-5.

## Introduction

Among the micronutrients, boron (B) and zinc (Zn) are cited as the most limiting worldwide for crop growth due to the low concentration of available nutrients in the soil solution and low contents in the soil rock parent material^1,2^. B is highly leached into the soil profile^3^, whereas Zn is highly adsorbed in the colloidal matrix of agricultural soils^4^. B and Zn play critical roles in plant physiology and biochemical processes, such as carbohydrate metabolism, protein synthesis, nucleic acid metabolism, and hormone regulation^5–7^. As such, B is an essential micronutrient for plant structure and rigidity and for forming a pectic network for cell adhesion by cross-linking pectic polysaccharide rhamnogalacturonans in the cell wall^7^. Zn is an essential nutrient that participates as a cofactor of over 300 enzymes, influencing the synthesis of the activity and structural integrity of proteins, nucleic acid biosynthesis, growth regulation, hormone balance, and chlorophyll formation^5,8–10^. A deficiency of B and Zn can cause nutritional disorders in plants, reducing the ability of the plants to grow and their adaptability to the environment^1,11^. Therefore, understanding the role of micronutrients in the plant and its physiological management is crucial for optimizing plant growth and ensuring robust crop production.

Under B deficiency conditions, lignin biosynthesis is reduced due to the impact of this nutrient on cell wall biosynthesis^12^. Xyloglucans are a type of hemicellulose found in plant cell walls. They are made of glucose and galactose molecules and cross-linked by borate esters, providing the cell wall strength and rigidity. Xyloglucan endotransglucosylase/hydrolases (XTHs) are enzymes involved in cell wall remodeling. The expression of specific XTH genes is influenced by B stress, reflecting alterations in cell wall properties^13,14^. Moreover, B imbalances can also induce oxidative stress in plants. Ascorbate peroxidase (APX) is involved in scavenging reactive oxygen species. B supplementation may be associated with changes in the activity of antioxidant enzymes, including APX, in safflower cultivars under different concentrations of H_3_BO_3_^15^; conversely, the upregulation of APX gene expression has also been connected to B deficiency and oxidative stress responses^16^. While B supplementation can enhance plant growth and stress tolerance, it is important to note that excessive B levels can lead to toxicity, disrupting cellular homeostasis and potentially exacerbating oxidative stress. The genes mentioned above may serve as markers of B deficiency in plants that play critical roles in various physiological processes affected by B availability.

Zn deficiency is prevalent worldwide, particularly in soils with specific characteristics such as sandy calcareous, high pH, high phosphorus content, or heavily weathered tropical soils^17^ due to factors like reduced solubility and availability due to the formation of insoluble Zn compounds. Zn deficiency can lead to various abnormalities, such as stunted plant growth, chlorosis of the younger leaves, and reduced crop yields. Addressing Zn deficiency through an improved understanding of its uptake, transport, and storage mechanisms in plants is critical for developing effective management strategies and ensuring sustainable agricultural practices. Therefore, proper management of Zn nutrition through fertilization is essential for addressing deficiencies and improving crop productivity.

Unlike many other nutrients, while mobile in the soil solution, B exhibits limited mobility within plant tissues. Roots primarily take it up as undissociated H_3_BO_3_, which moves passively with water through the xylem^7^. Membrane transporters mediate B translocation in plants^18–21^. In Arabidopsis, B transport is mediated primarily by two transporter families: Nod26-like Intrinsic Proteins (NIP) and anion exchangers (AE). The expression of NIP5;1, a key transporter involved in B uptake, is upregulated in root tissues under low B conditions to enhance B acquisition from the soil. Following uptake, B is transported to various plant tissues in a process facilitated by the anion exchanger BOR1, which is responsible for loading B into the xylem. The induction of BOR1 under B-deficient conditions ensures the effective long-distance transport and distribution of B throughout the plant. These coordinated responses are critical for optimizing B acquisition and internal distribution, particularly under conditions of limited B availability.

Conversely, under excess B conditions, the expression of these transporters is repressed to reduce nutrient uptake and toxicity. However, the expression patterns of B transporters in plants under high nutrient concentrations still require more detailed investigation. In this context, transcriptional control is a fundamental genetic mechanism by which plants regulate gene expression^22^. In contrast, additional mechanisms, such as post-transcriptional and translational regulation and protein modifications, usually act to fine-tune gene expression for precise cellular function. Previous studies have highlighted the importance of RNA-Seq technologies in advancing research on plant genomics. Tu et al. (2022) discussed the power of RNA-Seq beyond differential expression analysis, emphasizing the potential for exploring regulomics in plant research. Building upon this foundation of transcriptional control, our study investigates the critical roles of B and Zn in soybean physiology, with a particular emphasis on the transcriptional regulation of transporters and potential markers of nutrient status.

The uptake, translocation, and storage of Zn in plants are tightly regulated by a complex network of transporters and binding compounds^24^. Zn transporters (ZIP) are responsible for the uptake and distribution of this nutrient within plant cells, ensuring proper homeostasis^25^. These transporters facilitate the movement of Zn across biological membranes, maintaining intracellular and intra-organellar Zn homeostasis^26^. Beyond their role in homeostasis, Zn transporters play a crucial role in plant growth and development under stress conditions^27^. Studies have shown that the expression of Zn transporter genes in plants is influenced by the nutrient deficiency, indicating their adaptive response to low Zn environments^28^.

Foliar application is a recognized technique for directly supplying nutrients to plants, particularly during critical growth phases when soil nutrient availability is limited^1^. Absorption of nutrients via foliar fertilization in plants involves intricate mechanisms reliant on the leaf surface characteristics, specific nutrient uptake pathways, and subsequent physiological responses^29–31^. However, despite being critical, studies on these mechanisms at the genetic and molecular levels remain underexplored, leaving a significant gap in the current scientific literature. Plant genomics and transcriptomics present a promising avenue to unravel these complex processes, offering a potential blueprint for enhancing the efficiency of foliar fertilization. This knowledge is crucial for optimizing foliar fertilization strategies to improve nutrient uptake and plant growth and, ultimately, enhance crop productivity, contributing to sustainable agriculture and food security.

Foliar fertilizers containing micronutrients can be classified into different categories, including salts (such as sulfates, nitrates, and boric acid - H_3_BO_3_), chelates, solutions (for instance, B complexed with mono-ethylene-glycol), and suspension concentrates^30,32^. The salts, chelates, and solution are characterized as sources with low nutrient concentrations in their formulation. The form and application method of salts, chelates, and solutions often require lower nutrient concentrations to ensure effectiveness, stability, and safety in plant nutrient management. Suspension concentrate fertilizers are distinguished by their utilization of sparingly soluble sources, characterized by microparticles and a high concentration of nutrients. These fertilizers typically employ micronized oxides, carbonates, and hydroxides as nutrient sources^33^. Besides being utilized extensively in agriculture worldwide, there is still the need to enhance the understanding of these fertilizer sources on the molecular level of the plant and nutrient availability. Furthermore, there exists an opportunity to incorporate novel nutrient sources, such as zinc borate, a compound that chemically comprises two essential plant nutrients: B and Zn.

Zinc borate represents an underutilized source of Zn and B foliar supply in crop management. Investigations into the molecular physiology underpinning B and Zn nutrition in crops remain notably sparse. Moreover, despite its widespread application in agriculture, the molecular mechanisms governing nutrient assimilation via foliar fertilization have yet to be extensively explored. Here, we conducted global and targeted assessments of leaf expression profiles, following the foliar application of B and Zn (as zinc borate) in soybean plants under different nutrient regimens. This crop was chosen as a model due to its importance in the global economy^34^.

The transcriptional response to B was significantly more extensive than Zn, as evidenced by the number of differentially expressed genes following foliar fertilization. This study investigates the dynamic changes in gene expression in plants subjected to foliar fertilization with a suspension concentrate foliar fertilizer containing zinc borate as a nutrient source, with a particular emphasis on putative membrane transporters, such as NIP transporters, anion exchangers, and metal transporters. Another objective is to explore potential markers of B status in soybean plants. Additionally, this investigation aimed to simultaneously evaluate the transcriptional response to foliar supplementation with B and Zn when applied in combination. We conclude that zinc borate can be a potential source of these micronutrients in foliar applications, with B nutrition being the most affected.

## Materials and methods

### Plant material

The soybean seeds (*Glycine max* (L.) Merril) cv. Williams 82 (accession PI 518671) used in this study were obtained from the Soybean Germplasm Collection Station (Urbana, IL) of the USDA-Agriculture Research Service’s National Plant Germplasm System at the Germplasm Resources Information Network (GRIN).

### Growth conditions

Plants were cultivated on 5 × 1.5-m greenhouse benches in 1-gallon (3.8-L) plastic pots filled with washed silica sand (#4 Q-ROCK, US Silica). To ensure optimal growth conditions, the plants received ambient light supplemented with grow lights to maintain a 16-hour photoperiod during the initial 8 weeks of cultivation. Following this period, flowering was induced by removing light supplementation. The temperature was set to a constant 30 °C ± 2 °C throughout the experiment. Plants received complete Hoagland solution^35^ for the initial 2 weeks after sowing before applying the fertilization regimen for each treatment. The plants were fertilized to saturation twice weekly, with the sand medium being flushed with deionized (DI) water the day before each irrigation. Watering occurred as necessary between fertilizations. Plants were harvested 16 weeks after sowing.

### Treatments

According to each treatment, plants received full-strength modified Hoagland solution^35^ with specific amounts of B and/or Zn. Typical concentrations of micronutrients in the complete Hoagland solution include, besides other components, 46 µM (0.5 ppm) H_3_BO_3_ and 0.77 µM (0.05 ppm) zinc sulfate (ZnSO_4_). Treatments with low nutrient levels were set at 10% full-strength levels: 4.6 µM for low B and/or 0.077 µM for low Zn treatment.

### Experimental design

The experimental setup followed a randomized design, encompassing five treatments: (*i*) full-strength modified Hoagland solution (1X Hoagland) as a control; (*ii*) 1X Hoagland with low B (4.6 µM); (*iii*) 1X Hoagland with low Zn (0.77 µM); (*iv*) 1X Hoagland with low B and low Zn (4.6 µM and 0.77 µM, respectively); and (*v*) 1X Hoagland with low B and low Zn with foliar application at the calculated rates of 0.5–1.0 L/ha of product (as indicated below, considering a standing of 250,000 plants/ha). Application of the foliar fertilizer (zinc borate in a suspension concentration formulation from Yara International) consisted of a diluted solution of the suspension concentrate product containing 122 g/L B and 250 g/L Zn (active principle plus commercial wetting agents, adjuvants, and solvents). Plants were sprayed with foliar fertilizer at 4 and 8 weeks after sowing. Unless specified, tissues were collected 24 h after foliar fertilization had been applied.

### Experiment 1 (exp. 1)

This experiment aimed at capturing gene expression regulation by applying foliar fertilizer directly on the leaves analyzed. **Setup**: A subset of plants (*n* = 6) under low Zn/low B conditions received applications of the commercial zinc borate foliar fertilizer equivalent to 0.5 L of product per hectare (equivalent to 2 µL of fertilizer concentrate was applied per plant). For this experiment, the product was diluted in DI water (1:3,750 - typically, 800 µL diluted in 3 L), and 7.5 mL was sprayed over the whole plant) at 4th and 8th weeks after sowing (Sampling 1 and 2, respectively). Leaves directly treated with the product were sampled 1 day after applying the foliar fertilizer (4th and 8th weeks). Experiment 1 setup is more detailed in Suppl. Figure S1**.**

### Experiment 2 (exp. 2)

In this experiment, our goal was to identify genes induced systemically by foliar fertilizer applied to more basal tissues of the plant. **Setup**: A subset of plants (*n* = 6 per treatment) under low Zn/low B conditions that had not received any foliar fertilization until the 8th week after sowing were applied foliar fertilizer equivalent to 1.0 L of product per hectare. Plant material in the next phytomer (leaf and stem above the region where the foliar fertilizer was applied) was collected one week after the application of the product. Control plants were kept under low Zn/low B conditions and did not receive applications of the commercial foliar formulation, and samples were collected simultaneously. Experiment 2 setup is shown in more detail in Suppl. Figure S2**.**

### Experiment 3 (exp. 3)

We aimed to gain insights into nutrient uptake mechanisms after foliar fertilization in plants undergoing B and Zn deprivation. Setup: A subset of plants (*n* = 6 per treatment) under a low Zn/low B regimen without any foliar fertilization received the first application of the commercial foliar fertilizer equivalent to 1.0 L of product per hectare at the 8th week after sowing. Leaves directly treated with the product were sampled one day after application. Leaves that did not receive any foliar fertilization were sampled as controls. Experiment 3 setup is shown in Suppl. Figure S3.

### Agronomic parameters and statistical analyses

The weight of dry seeds (per plant), the number of pods per plant, and the number of seeds per plant were recorded upon harvest. Data were tested for normality using the Shapiro-Wilk test and, when needed, log-transformed and re-tested for normality. Factors and interactions were assessed with Analysis of Variance (ANOVA) followed by a post-hoc Tukey’s multiple range test. Significant differences were noted for *P*-value ≤ 0.05. The graphs and statistical analyses were generated using GraphPad Prism version 9.00 for Mac (GraphPad Software, La Jolla, CA, USA).

### Sampling and material processing

For Exps 1 and 3, plant materials were collected 24 h after foliar fertilization for molecular analyses. The youngest fully expanded leaves were collected from each plant, and leaflets were individually placed into Falcon tubes, snap-frozen in liquid nitrogen, and kept at − 80 °C until processing. For Exp. 2, leaf and stem materials adjacent to the application area (the next younger phytomer) were collected one week after foliar fertilization in the same way as described previously. Plants from which tissues were sampled were subsequently discarded.

### RNA isolation and mRNA-Sequencing

One leaflet per plant was ground manually using a pestle and mortar. RNA was subsequently isolated using the mirVana miRNA isolation kit (AM1560, ThermoFisher Scientific), following the fabricant’s recommendation for total RNA extraction. Samples were quantified in NanoDrop ND-1000 Spectrophotometer (ThermoFisher Scientific), and the quality was assessed in BioAnalyzer 2100 (Agilent). Library preparation and sequencing were carried out on an Illumina NovaSeq 6000 sequencing system by Novogene (Sacramento, CA) using a PE150 strategy and a depth of 20 M paired reads per sample.

### Mapping of reads and DEG analysis

High-quality reads were mapped to the reference *G. max* genome sequence Wm82.a4.v1 (www.phytozome-next.jgi.doe.gov) using STAR v.2.7.10a^36^ with default settings. The BAM files of uniquely mapped reads were inputs for StringTie v.2.2.1 ^37^ and FeatureCounts v. 2.0.1 ^38^ programs. Gene expression was estimated using FPKM for statistical analyses and converted as TPM for data visualization and interpretation. Comparisons of gene expression with adjusted *P*-value ≤ 0.05 were carried out using DEseq v.2 ^39^. Subsequently, to consider a gene differentially expressed (DEG), the parameters used were adjusted *p*-value ≤ 0.05, log2 fold-change ≥ |2| between treatments, and a normalized minimum mean expression (FPKM) ≥ 2 in at least one treatment.

### Elemental analysis

A matching set of the plant material (leaflets) used for RNA-Seq was sent to Yara’s MegaLabs for ICP (inductively coupled plasma) spectroscopy analysis. Plant material was freeze-dried for 7 days. The samples were crushed using a mill and sieved through a 20 mesh for elemental composition analyses. For quantifying levels of phosphorus (P), potassium (K), calcium (Ca), magnesium (Mg), sulfur (S), copper (Cu), Fe, manganese (Mn), molybdenum (Mo), Zn, and B, the samples were subjected to a nitric perchloric solution (65% and 70%), with the resulting extract being analyzed with an inductively coupled plasma optical emission spectrometer (ICP-OES, Perkin-Elmer Optima 3000XL).

## Results and discussion

### Plant development and nutritional status

Plant development was similar across all treatment groups (Suppl. Figure S4), with no significant differences in yield (expressed as the number of pods, number of seeds, and total seed weight) according to Tukey’s test at the 95% confidence level (Suppl. Figure S5). These findings imply that the B and Zn concentrations supplied in the low nutrient conditions (comprising 10% of the Hoagland solution recipe for either or both nutrients) in the sand substrate and the quantity of these nutrients in the seeds were sufficient to support the development of soybean plants. Next, we conducted further analyses to determine whether the low-nutrient treatments and foliar fertilization could have induced detectable molecular responses at the gene expression level.

**Fig. 1:**
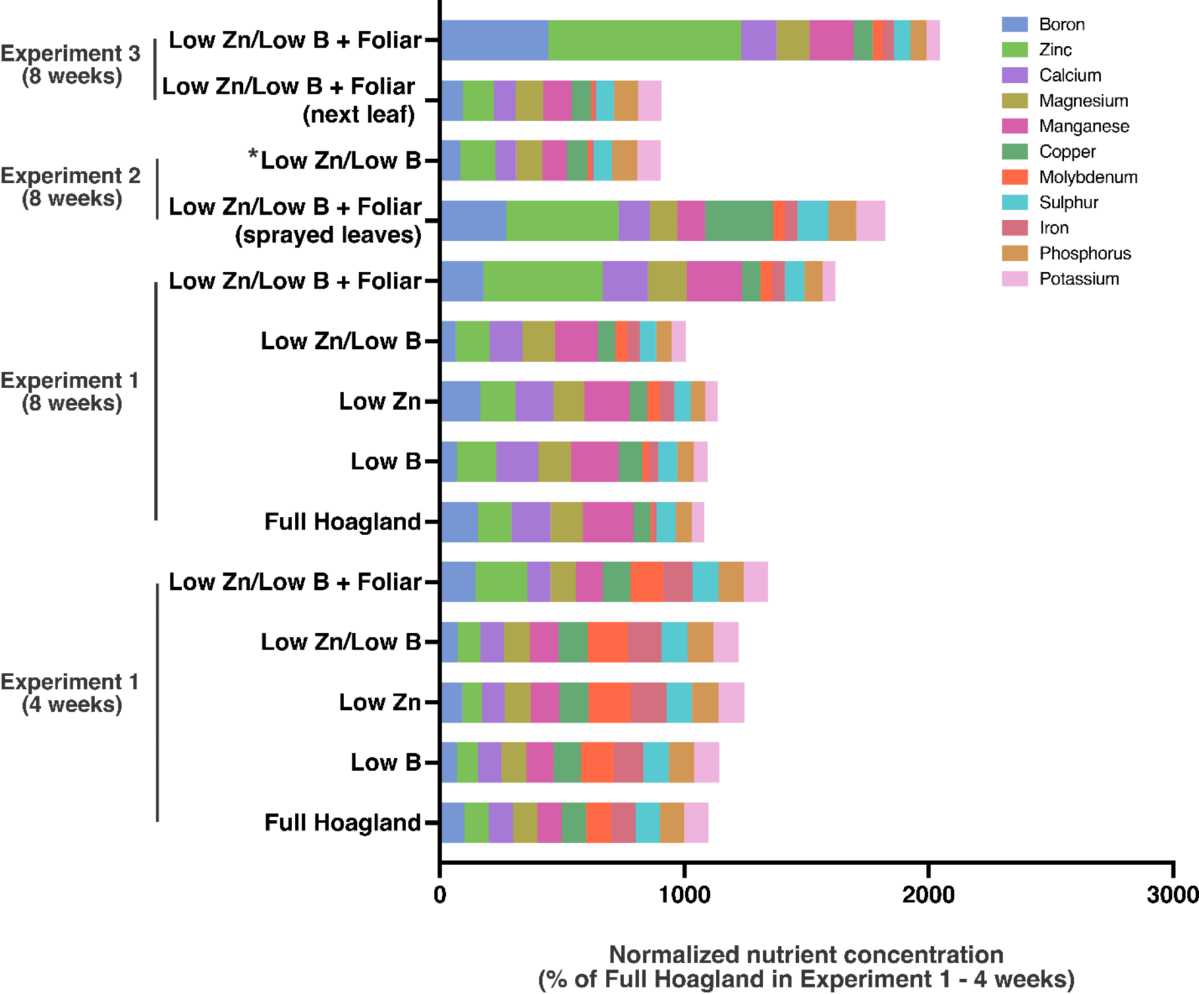
Nutritional composition of soybean leaves. The values are means of three biological replicates (parts per million) that were normalized considering the treatment Full Hoagland solution (Experiment 1 – Sampling 1 at 4 weeks after sowing) as a baseline. *This control group was also used for Experiment 3. The mineral content for all experiments was normalized to the complete Hoagland control in Experiment 1, which was set at 100% for each mineral analyzed.

In our study, we observed diverse responses across the experiments and treatments regarding the nutritional composition of soybean leaves, as detailed in Fig. 1. Initially, in Exp. 1, no significant differences were noted in macronutrient levels (P, K, S, Ca, and Mg) 4 weeks after sowing, indicating minimal impact of the treatments. However, more pronounced changes were evident in the 8-week samples, particularly in treatments combining low Zn/low B conditions. Notably, foliar applications led to increased levels of B and Zn, suggesting the potential of foliar fertilization to mitigate mineral deficiencies over extended periods.

By the same token, 8 weeks after the sowing also revealed significant increases in sulfur (S), calcium (Ca), and magnesium (Mg) in plants receiving foliar fertilization compared to the low Zn/low B controls in all three experiments. This highlights a substantial response and emphasizes the roles that S, Ca, and Mg, often categorized as secondary macronutrients, play pivotal roles in plant nutrition but are frequently underestimated by crop producers^40^. In Exp. 2, increased levels of K, S, and Ca were observed in the sprayed leaves compared to those from the low Zn/low B treatment, illustrating localized nutrient uptake. Exp. 3 further demonstrated variable responses in all macronutrients but S, attributed to the immediate effects of foliar applications on soybean leaves^41^. Similar trends were observed with micronutrients, where B levels increased in all foliar-treated plants^42,43^. Zn also showed increased levels in all foliar treatments, mirroring the macronutrient patterns. Notably, except for copper (Cu), variations in all micronutrients were more pronounced in Exp. 3, indicating the significant influence of direct foliar nutrient delivery on plant nutritional status. We also detected a significant positive correlation between Zn and Mg (Spearman’s rank correlation coefficient, *ρ* = 0.56, *p*-value = 0.037) when analyzing all samples together. All other correlations between Zn or B with the other elements were not significant (*p*-value ≤ 0.05, data not shown).

### Global transcriptional analyses

**Fig. 2:**
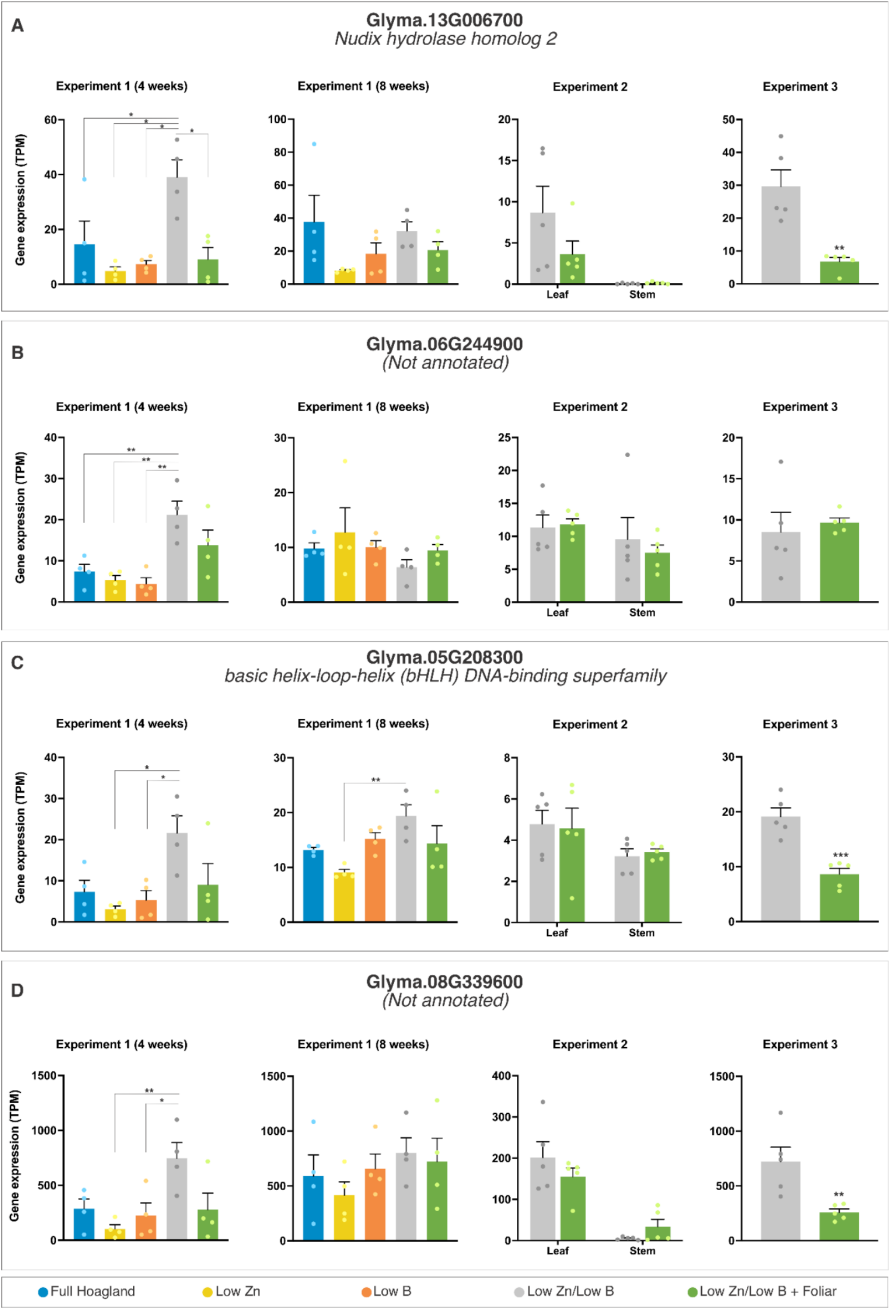
Transcriptional profiles of representative differently expressed genes (DEGs) in Experiment 1 Sampling 1 (4 weeks). The data are means ± SEM (*n* ≥ 4 biological replicates) of read counts normalized in transcript per million reads (TPM) for each experimental setup. The asterisks indicate a significant difference between the groups according to Tukey’s test at *p* < 0.05 (*), *p* < 0.01 (**), and *p* < 0.001 (***).

The complete dataset with normalized gene expression values for individual samples in this study is available in Suppl. Table S1. We also provide searchable tables with the statistical analyses of each experiment comparison of DEGs to allow the reader to explore the dataset (Suppl. Datasets S1-S4). Upon examination of the expression profiles of the 52,872 soybean genes annotated using stringent criteria (*p*-adj ≤ 0.05, log2 fold change ≥ |2|, and mean FPKM ≥ 2 in at least one treatment), we observed no differentially expressed genes (DEGs) under our experimental conditions when comparing leaves from 4-week-old plants treated with low Zn or low B Hoagland solution to those with a complete nutrient solution, 24 h post-application of foliar fertilizer (Exp. 1, Sampling 1: Table 1, Suppl. Data S1). The absence of DEGs could be attributed to either the presence of residual levels of Zn in the growth medium or the sufficiency of either 0.77 µM Zn or 4.6 µM B in the fertilizer solution for root uptake and nutrient mobilization. Notably, the comparisons between the combined treatment with low Zn/low B against low dosages of individual nutrients, either B or Zn, at 4 weeks of cultivation yielded the highest number of DEGs, with 57 and 300 genes, respectively (Exp. 1, Sampling 1) (Table 1, Suppl. Data S1). A gene annotated as Nudix hydrolase homolog (Glyma.13G006700, depicted in Fig. 2A) was identified as a differentially expressed gene (DEG) along with 40 other genes across both datasets analyzed. The consistent upregulation of this gene suggests a potential role in maintaining cellular homeostasis and mitigating stress effects associated with low-nutrient availability during the early stages of plant development. Other genes displaying similar expression patterns include a basic helix-loop-helix (bHLH) transcription factor (Glyma.05G208300) and two genes lacking functional annotations (Fig. 2B–D). These genes may serve as promising candidates for the early detection of nutritional deficiencies, and their functions may explain broader aspects of plant stress physiology and adaptation strategies. Interestingly, only 10 DEGs were detected when comparing the low Zn/low B control with the group that received foliar fertilization at 4 weeks, suggesting that the foliar treatment alleviated the effects of combined nutrient deficiencies.

**Table 1:** The number of significantly differentially expressed genes (DEGs) between treatments in pairwise comparisons. Filters used: *p*adj ≤ 0.05, log2 fold-change ≥ |2|, mean FPKM ≥ 2 in at least one treatment.

Pairwise contrast	Experiment 1	Experiment 2	Experiment 3
	Sampling 1	Sampling 2	Leaf Stem Leaf
Full vs. foliar	4	162	- - -
Full vs. lowZn/lowB	4	153	- - -
Full vs. lowB	0	32	- - -
Full vs. lowZn	0	68	- - -
Foliar vs. lowZn/lowB	10	1	134 338 530
Foliar vs. low B	1	1	- - -
Foliar vs. low Zn	4	136	- - -
lowB vs. lowZn	1	44	- - -
lowB vs. lowZn/lowB	57	1	- - -
lowZn vs. lowZn/lowB	300	167	- - -

See the material and methods section for details on experimental setup and statistical analyses of RNA-Seq data. For Experiment 1, Samplings occurred 24 h after applying foliar fertilizer at 4 and 8 weeks after sowing for sampling 1 and 2. Supplemental Data files S1-S4 contain tables with DEGs and the statistics of each comparison for each subset of experiments. Dashes indicate contrasts were not carried out.

**Fig. 3:**
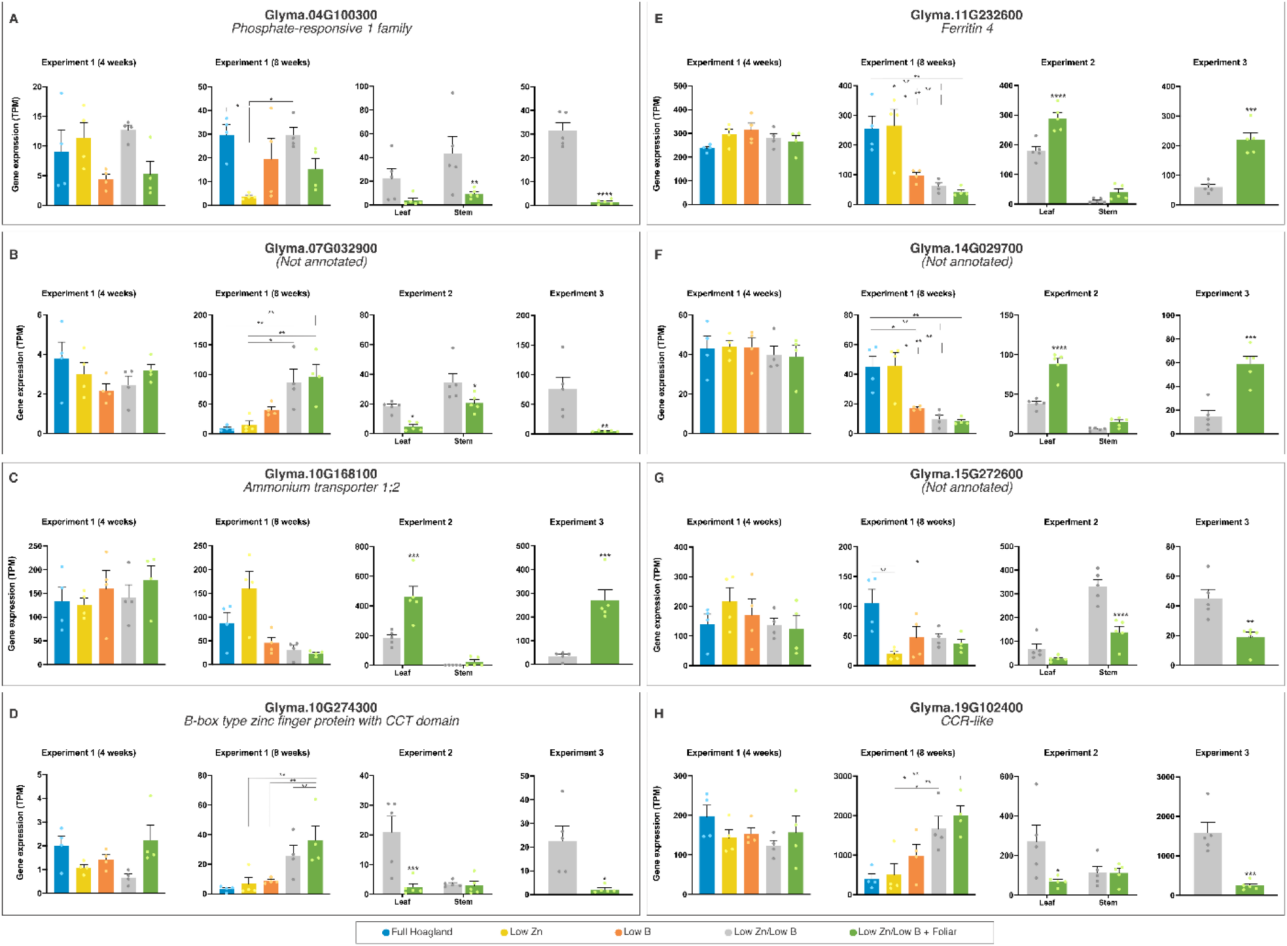
Transcriptional profiles of representative differently expressed genes (DEGs) in Experiment 1 Sampling 2 (8 weeks). The data are means ± SEM (*n* ≥ 4 biological replicates) of read counts normalized in transcript per million reads (TPM) for each experimental setup. The asterisks indicate a significant difference between the groups according to Tukey’s test at *p* < 0.05 (*), *p* < 0.01 (**), *p* < 0.001 (***), and *p* < 0.0001 (****).

In week 8 (Exp. 1, Sampling 2), we identified 32 DEGs for the contrast between low B treatment and the complete Hoagland solution (Table 2, Suppl. Data S2), eight of which lacked functional annotation (three depicted in Fig. 3). In this same comparison, 10 repressed genes under low B (from 6 up to 52-fold) are annotated as heat-shock proteins (HSPs), which have been implicated with diverse stresses in soybean^44^. However, under low nutrient conditions, plants may repress the expression of HSPs to conserve resources for critical processes, reflecting a strategic reallocation that prioritizes nutrient uptake and essential metabolic functions over broad-spectrum stress responses. This adaptation highlights the complex regulatory trade-offs plants manage in response to nutrient insufficiency, potentially influencing their resilience to multiple stresses (Table 3).

**Table 2:** Genes identified coding for potential boron transporters in the soybean genome.

Gene ID	Protein length (aa)	Top BlastP Hit in Arabidopsis (TAIR10)	Evalue
IPR011531: HCO_3_^−^ cotransporters (putative BOR effluxers)			
Glyma.03g222300	724	At3g62270|BOR2	0
Glyma.04g069500	663	At1g15460|BOR4	0
Glyma.04g184000	721	At3g62270|BOR2	0
Glyma.05g038300	681	At3g62270|BOR2	0
Glyma.06g071200	656	At1g15460| BOR4	0
Glyma.06g181900	724	At3g62270|BOR2	0
Glyma.09g031400	724	At3g62270|BOR2	0
Glyma.13g362550 Y	122	At1g15460| BOR4	1e−51
Glyma.13g362600	510	At1g15460| BOR4	0
Glyma.15g011200	670	At1g15460| BOR4	0
Glyma.15g136466 Y	124	At2g47160|BOR1	7e−59
Glyma.15g136532 Y	234	At3g62270|BOR2	1e−93
Glyma.17g088800	653	At3g62270|BOR2	0
Glyma.17g207700	664	At5g25430	0
Glyma.19g219500	724	At3g62270|BOR2	0
IPR000425: Nod26-like aquaporins (putative NIP channels)			
Glyma.02g140500	297	At3g06100.1 | NIP7;1	1e−90
Glyma.02g246700	262	At5g37820.1 | NIP4;2	2e−114
Glyma.05g162500	272	At4g18910.1 | NIP1;2	2e−131
Glyma.05g162600	271	At4g18910.1 | NIP1;2	3e−133
Glyma.07g024400	218	At1g80760.1 | NIP6;1	2e−91
Glyma.07g024700	185	At1g80760.1 | NIP6;1	2e−73
Glyma.07g026900	184	At1g80760.1 | NIP6;1	5e−73
Glyma.07g217700	263	At5g37810.1 | NIP4;1	3e−103
Glyma.08g120100	272	At4g18910.1 | NIP1;2	2e−126
Glyma.08g120200	275	At4g18910.1 | NIP1;2	6e−135
Glyma.08g217400	307	At1g80760.1 | NIP6;1	4e−157
Glyma.09g238200	294	At5g37820.1 | NIP4;2	1e−65
Glyma.10g221100	300	At4g10380.1 | NIP5;1	2e−148
Glyma.13g089300	252	At4g18910.1 | NIP1;2	5e−49
Glyma.13g224900	274	At4g18910.1 | NIP1;2	1e−134
Glyma.14g069501 Y	134	At5g37820.1 | NIP4;2	8e−57
Glyma.14g109600 Y	105	At1g80760.1 | NIP6;1	1e−49
Glyma.14g174300	271	At4g18910.1 | NIP1;2	1e−67
Glyma.15g003900	305	At1g80760.1 | NIP6;1	2e−163
Glyma.15g087300	274	At4g18910.1 | NIP1;2	2e−132
Glyma.18g259500	296	At5g37820.1 | NIP4;2	6e−65
Glyma.20g170400	300	At4g10380.1 | NIP5;1	3e−150

Genes in gray font followed by the symbol Y are potential pseudogenes with truncated coding sequences. Supplemental Table 1 provides more details on the annotation of putative gene products.

**Table 3:** Selected genes identified coding for potential zinc transporters in the soybean genome.

Gene ID	Protein length (aa)	Top BlastP hit in arabidopsis (TAIR10)	Evalue
IPR003689: ZIP metal ion transporters			
Glyma.08G092700	486	AT1G68100.1 | IAR1	1e−143
Glyma.09G271900	599	AT3G08650.2 | ZIP	0
Glyma.11G169300	327	AT2G30080.1 | ZIP6	2e−149
Glyma.13G340900	277	AT3G20870.1 | ZIP29	4e−159
Glyma.15G033500	283	AT3G20870.1 | ZIP29	2e−139
Glyma.18G217100	599	AT3G08650.2 | ZIP	0
IPR006121: HMT/detox ATPases (heavy metal associated proteins)			
Glyma.02G222600	315	AT5G24580.2 | ATHMP47	5e−76
Glyma.03G212400	345	AT5G27690.1 | ATHMP49	2e−23
Glyma.04G106500 Y	150	AT4G08570.1 | ATHMP33	4e−81
Glyma.04G197300	320	AT5G50740.1 | ATHMP52	2e−77
Glyma.05G098800	833	AT4G37270.1 | ATHMA1	0
Glyma.05G181500	154	AT4G39700.1 | ATHMA15	5e−67
Glyma.05G203700	309	At5g50740.1 | ATHMP52	3e−72
Glyma.05G211600	161	At1g01490.2 | ATHMP01	1e−23
Glyma.06G106700 Y	150	At4g08570.1 | ATHMP33	2e−81
Glyma.06G168300	332	At4g08570.1 | ATHMP33	2e−76
Glyma.07G065800 Y	152	At1g01490.2 | ATHMP01	4e−17
Glyma.07G257300	491	At3g06130.2 | ATHMP25	5e−41
Glyma.08G011100	311	At5g63530.2 | FP3	2e−60
Glyma.09G013700	242	At4g16380.1 | ATHMP09	9e−18
Glyma.09G130400 Y	151	At1g01490.2 | ATHMP01	9e−11
Glyma.09G255100 Y	181	At1g01490.2 | ATHMP01	2e−30
Glyma.10G055300	323	At5g03380.1 | ATHMP43	7e−45
Glyma.10G156100	561	At3g06130.2 | ATHMP25	1e−31
Glyma.10G171800 Y	179	At2g18196.1 | ATHMP16	2e−85
Glyma.11G194600	323	At5g60800.1 | ATHMP53	4e−47
Glyma.11G238000 Y	156	At4g39700.1 | ATHMP41	2e−62
Glyma.12G079500	297	At5g60800.1 | ATHMP53	8e−42
Glyma.13G070100 Y	186	At5g14910.1 | HMP	9e−39
Glyma.13G099600	1097	At4g30110.1 | HMA2	0
Glyma.13G142400	332	At2g36950.1 | ATHMP20	1e−35
Glyma.13G208400	504	At3g06130.2 | ATHMP25	4e−41
Glyma.14G189500	320	At5g24580.2 | ATHMP47	2e−78
Glyma.15G104400	493	At3g06130.2 | ATHMP25	3e−41
Glyma.15G118400	248	At4g16380.1 | ATHMP09	1e−21
Glyma.15G118500	268	At5g50740.1 | ATHMP52	7e−45
Glyma.16G032400 Y	154	At1g01490.2 | ATHMP01	1e−15
Glyma.17G006200	263	At5g50740.1 | ATHMP52	2e−50
Glyma.17G006300	260	At4g16380.1 | ATHMP09	2e−18
Glyma.17G016800	500	At3g06130.2 | ATHMP25	1e−41
Glyma.17G166800	818	At4g37270.1 | ATHMA1	0
Glyma.18G019300 Y	158	At4g39700.1 | ATHMP41	1e−57
Glyma.18G238000 Y	177	At1g01490.2 | ATHMP01	1e−31
Glyma.19G012900	237	At5g14910.1 | HMP	2e−37
Glyma.19G184100	379	At4g16380.1 | ATHMP09	2e−45
Glyma.19G209500	295	At5g27690.1 | ATHMP49	1e−25
Glyma.20G218500 Y	179	At2g18196.1 | ATHMP16	1e−84
Glyma.20G232200	531	At3g06130.2 | ATHMP25	1e−32
IPR08217: VIT family			
Glyma.02G220500	977	At4g27860.2 | MEB1	1e−40
Glyma.05G240600	248	At2g01770.1 | VIT1	4e−129
Glyma.08G181900	218	At3g43660.1 | NOD21/VIT	2e−81
Glyma.10G225900	223	At3g43660.1 | NOD21/VIT	1e−83
Glyma.16G168200	234	At3g43660.1 | NOD21/VIT	1e−91
Glyma.20G166100	222	At3g43660.1 | NOD21/VIT	1e−91
IPR001046: NRAMP metal ion transporters			
Glyma.06G044200	523	At1g47240.1 | NRAMP2	0
Glyma.10G058300	1326	At5g03280.1 | EIN2/CKR1	0
Glyma.13G145100	1314	At5g03280.1 | EIN2/CKR1	0
Glyma.15G003500	547	At1g15960.1 | NRAMP6	0
IPR004698: Fe-regulated transporters			
Glyma.06G052000	479	At1g60960.1 | IRT3	6e−168
Glyma.17G228600	394	At1g10970.1 | ZIP4	9e−88

Genes in gray font followed by the symbol Y are potential pseudogenes with truncated coding sequences (≥ 200 amino acid residues) and minimal expression (≥ 20 TPM in at least one sample). Supplemental Table S1 provides more details on the annotation of putative gene products. Supplemental Data S5 provides the complete list of genes from potential zinc transporter families in the soybean genome.

Additional differentially expressed genes (DEGs) identified in Exp. 1 – Sampling 2 included a phosphate-responsive protein, a ferritin, and an ammonium transporter, reflecting changes in the overall nutritional status of the plants caused by low supply of micronutrients. Furthermore, among the DEGs, we identified a potential transcription factor (B-box zinc finger protein) and a CCR-like protein, both of which are critical for modulating lignin content and composition (Fig. 3: notice also the significant expression changes in Exp. 2 and 3).

**Fig. 4:**
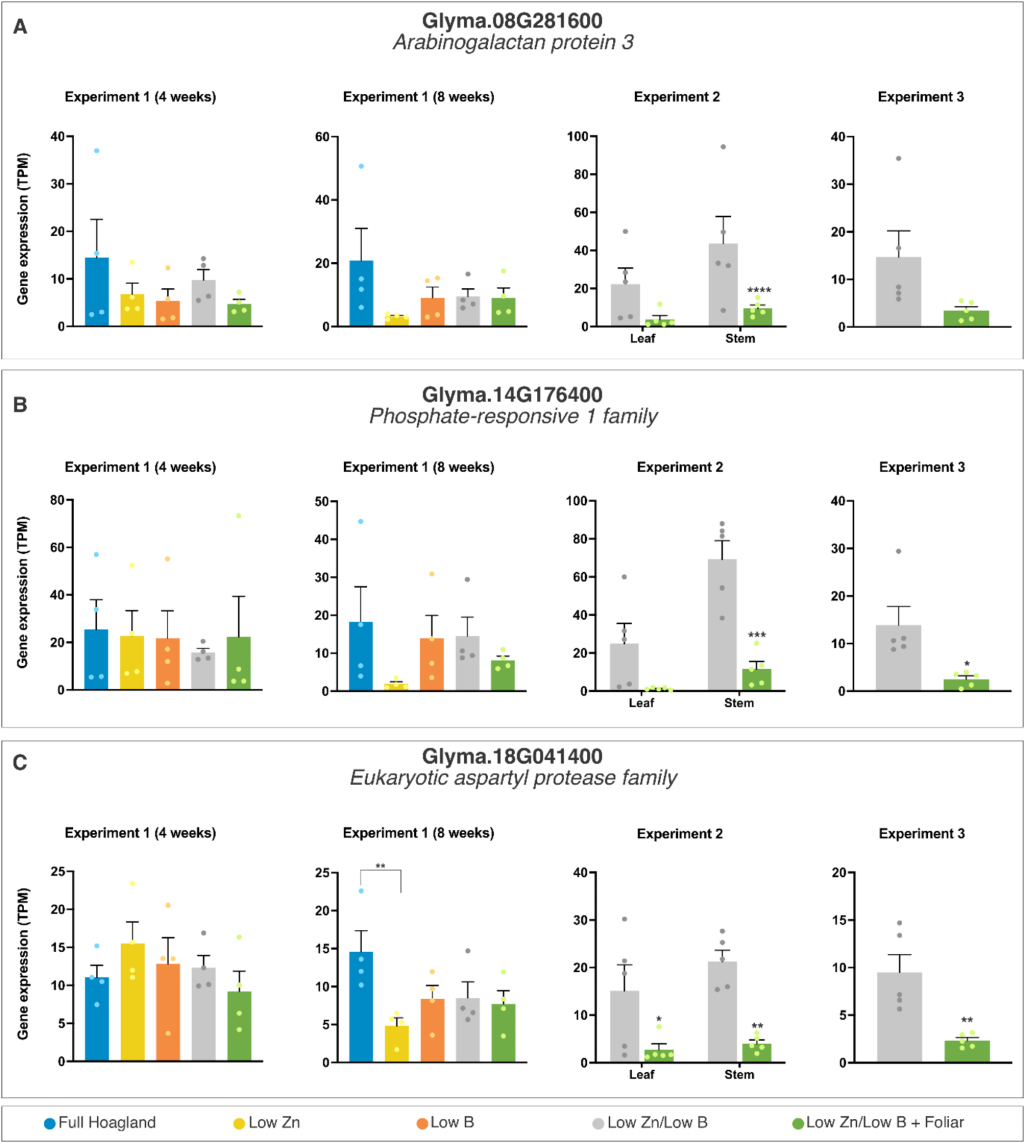
Transcriptional profiles of representative differently expressed genes (DEGs) in Experiment 2. The data are means ± SEM (*n* ≥ 4 biological replicates) of read counts normalized in transcript per million reads (TPM) for each experimental setup. The asterisks indicate a significant difference between the groups according to Tukey’s test at *p* < 0.05 (*), *p* < 0.01 (**), *p* < 0.001 (***), and *p* < 0.0001 (****).

We also compared leaves and stems above the region that received 1 L/ha foliar fertilization for the first time at week 8 in plants growing under low Zn/low B conditions (Exp. 2) to tissues from plants that did not receive any foliar application. We identified 134 DEGs in the leaves and 338 in the stems in samples harvested 1 week after plants had received foliar fertilization (Table 1, Suppl. Data S3). The expression of these genes was systemically regulated since the tissues analyzed were adjacent to the application site and did not receive fertilization directly. Thirty-two genes appeared as DEG in both tissues, including an arabinogalactan protein (Glyma.08G281600), a member of the phosphate-responsive 1 family (Glyma.14G176400), and a eukaryotic aspartyl protease (Glyma.18G041400) (Fig. 4). All differentially expressed genes (DEGs) identified in Exp. 2 exhibited significant downregulation following foliar fertilization in the neighboring leaves and stems. The observed repression of numerous genes associated with cell wall metabolism - including arabinogalactans, xyloglucan endotransglucosylase/hydrolases (XTHs), and various biosynthesis enzymes - as well as genes involved in membrane transport, such as ammonium channels, phosphate transporters, and heavy metal transporters/detoxification proteins, suggests a potential alleviation of stress within the system due to the foliar fertilization.

**Fig. 5:**
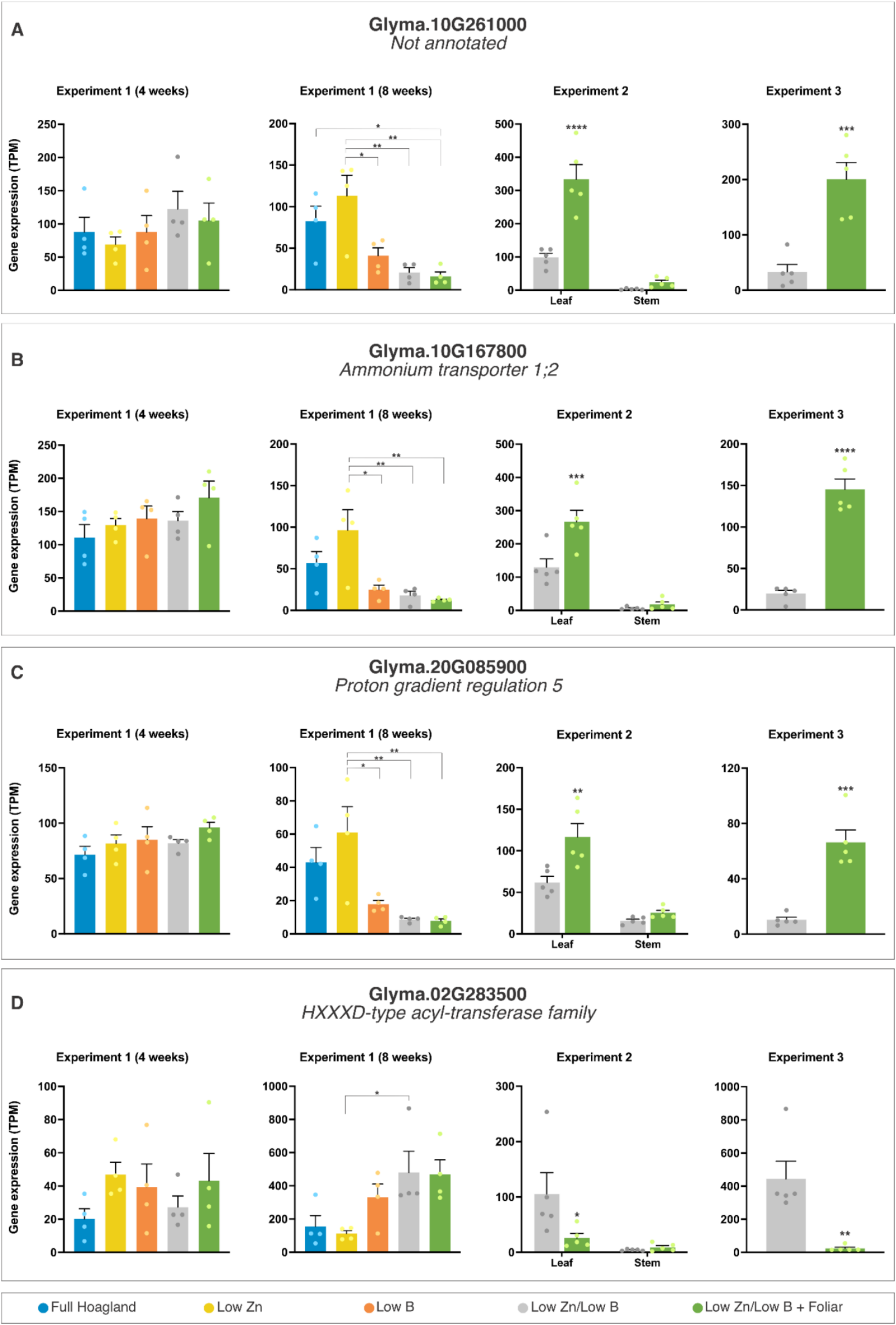
Transcriptional profiles of representative differently expressed genes (DEGs) in Experiment 3. The data are means ± SEM (*n* ≥ 4 biological replicates) of read counts normalized in transcript per million reads (TPM) for each experimental setup. The asterisks indicate a significant difference between the groups according to Tukey’s test at *p* < 0.05 (*), *p* < 0.01 (**), *p* < 0.001 (***), and *p* < 0.0001 (****).

The gene expression profile in Exp. 3 differs drastically from that observed in Exp. 2, not only in the total number of differentially expressed genes identified (530: Table 1) but also in the nature and extent of transcriptional regulation (326 genes repressed and 204 induced: Suppl. Data S4). These differences can be attributed to the direct foliar fertilization of the tissues in Exp. 3, with leaves collected one day post-application, compared to one week in Exp. 2. Figure 5 provides some examples of DEGs identified in Exp. 3. Distinct functional annotations were predominantly associated with the downregulated differentially expressed genes (DEGs) from Exp. 3, including C_2_H_2_ and C_2_HC zinc fingers (6 genes), calmodulin- and calcium-binding domain proteins (14), cysteine-rich receptor-like kinases (4), disease resistance-responsive proteins (6), and heavy metal transport/detoxification proteins, among others. Conversely, categories significantly represented among the DEGs induced by foliar application featured UDP-glycosyl transferases (6), spermidine hydroxycinnamoyl transferases, cytochrome P450 polypeptides, B-box zinc fingers (8), and ammonium transporters (3), along with genes essential to photosynthesis such as chlorophyll A-B binding proteins (4), fructose-bisphosphate aldolases (3), magnesium-chelatases (2), and Rubisco activase (4). These findings underscore the diverse regulatory impacts of direct foliar fertilization on gene expression, highlighting suppressive and promotive effects on key functional genes. The findings from Exp. 3 may indicate new mechanisms of nutrient assimilation and metabolism that are activated during foliar feeding.

### Transcriptional modulation by B supply

The modulation of transcriptional activity resulting from foliar-supplied B was apparent in the expression profiles of potential transporters and genes indicative of low B status. We conducted a comprehensive survey of the soybean genome to identify genes within families reported to transport boric acid. Additionally, we investigated genes that are regulated by B to identify potential markers of its status within the plant.

#### Identification of B transporters in the soybean genome

Boron effluxes (BOR) functionally characterized in Arabidopsis belong to the AE family (Transporter Classification Database TCDB #2.A.31, InterPro bicarbonate transporter motif IPR003020/Pfam PF00955). In angiosperms, BOR transporters are divided into two clades representing distinct archetypes and roles in B homeostasis: clade I includes AtBOR1, AtBOR2, and AtBOR3. AtBOR1 is expressed in the pericycle cells around the xylem of roots and shoots. It is required for B uptake and loading in roots and the efficient translocation of B into shoots under low B conditions^45,46^. AtBOR2 is necessary for distribution in the wall of root cells, allowing root elongation under limited B conditions^47^. AtBOR4 exporter is the clade II archetype. It protects the cells against B toxicity^48^ by increasing its expression under high B conditions^49^. Clade II transporters also include AtBOR5, AtBOR6, and AtBOR7. Here, we identified 15 soybean genes annotated as HCO_3_^−^ cotransporters, equivalent to the TCDB anion exchanger family (Table 2). Despite their official categorization as bicarbonate transporters, all plant proteins grouped within this family in the TCDB database exhibit characteristics consistent with B transporters, forming a distinct subfamily (TCDB #2.A.31.3) labeled as B effluxes within this classification system. Three of these genes show a premature stop codon resulting in short proteins (< 250 amino acid residues) and likely are pseudogenes. Therefore, 12 genes in the soybean genome potentially code for functional BOR transporters. This family size in the soybean genome is similar to the wheat genome (*Triticum aestivum* L.), which is another polyploid crop that contains 14 BOR genes^50^.

Subclass-II NIP channels (NIP5;1, NIP6;1, and NIP7;1) facilitate the influx of H_3_BO_3_ in Arabidopsis^51^. They belong to the larger NIP family (TCDB #1.A.8.12, InterPro motifs IPR000425/PF00230 and aquaporin-like IPR023271), which are part of the Major Intrinsic Protein (MIP) superfamily of aquaporins. In Arabidopsis, AtNIP5;1 is critical for H_3_BO_3_ to pass through the root epidermis^52^. MtNIP5;1 (Medtr1g097840) has been characterized in the model legume *Medicago truncatula* as a functional homolog of the AtNIP5;1 ^53^. AtNIP6;1 is expressed mainly in the phloem of stem nodes, significantly contributing to the translocation of B to young, expanding leaves^54^. AtNIP7;1 is prominently expressed within the tapetum cells of anthers during flower development, suggesting a role in reproduction^55^. Moreover, AtNIP1;1 is a promiscuous aquaporin with an affinity to H_3_BO_3_ in addition to water, arsenic, silicon, glycerol, urea, ammonia, and lactic acid^56–58^. We identified 22 genes coding for NIP proteins, 2 of which may be too short (< 150 amino acid residues) for adequately carrying out their molecular functions. Thus, 20 genes code for NIP transporters with potential affinity to boric acid in the tissues of the soybean plant (Table 2).

#### Potentially relevant membrane transporters regulated by B nutritional status

**Fig. 6:**
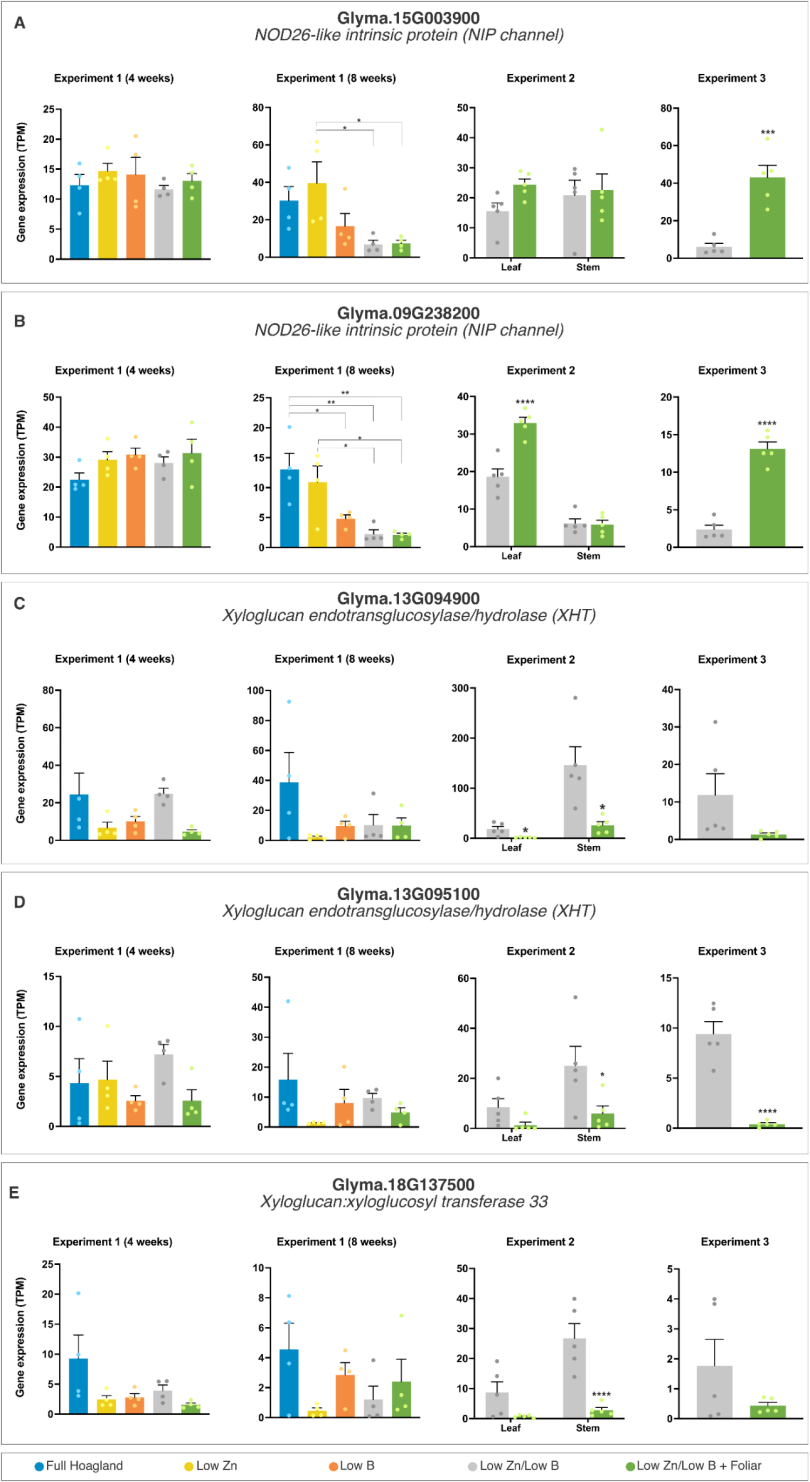
Transcriptional profiles of differently expressed genes (DEGs) regulated by boron supply across treatments as potential indicators of boron status in the plant. The data are means ± SEM (*n* ≥ 4 biological replicates) of read counts normalized in transcript per million reads (TPM) for each experimental setup. The asterisks indicate a significant difference between the groups according to Tukey’s test at *p* < 0.05 (*), *p* < 0.01 (**), *p* < 0.001 (***), and *p* < 0.0001 (****).

The regulation of membrane transporters by B nutritional status is underscored by the differential expression of the transporters NIP5;1 and NIP6;1. In Arabidopsis, NIP5;1 is located primarily in the root elongation zones and root hairs. It is markedly upregulated in response to B deficiency^52^. Contrastingly, NIP6;1 expression in shoots plays a critical role in B mobilization within the vascular system and in supplying B to meristematic regions^54^. This differential expression pattern underlines the finely tuned plant response to variations in B availability, reflecting the critical role of NIP channels in maintaining B homeostasis. Among the NIP channels in soybean (Suppl. Figure S6), remarkable expression patterns emerged, particularly for Glyma.15G003900 and Glyma.09G238200, which showed significant upregulation following foliar application in Exp. 3 (Fig. 6A,B). The major intrinsic protein (MIP) NtXIP1;1 channel in *Nicotiana tabacum* is specific for H_3_BO_3_ and localizes in the epidermal tissues of roots and shoots. NtXIP1;1 expression is upregulated under B-limited conditions. In turn, its overexpression caused B deficiency symptoms that could be alleviated by B fertilization^59^. A homologous gene in soybean, Glyma.12G023600, was identified but did not show significant expression changes under our experimental conditions, suggesting species-specific responses to B stress. This may indicate different regulatory mechanisms for B homeostasis or that a threshold of B deficiency was not fully achieved in our study to trigger a stronger response.

#### Stress indicators of B status

Within the array of genes responsive to B availability, some have emerged as potential biomarkers for assessing the B nutritional status of plants (Fig. 6, Suppl. Fig. S7). For example, Glyma.18G137500, a gene encoding a putative xyloglucan: xyloglucosyl transferase (XXT) implicated in xyloglucan biosynthesis and cell wall structure^60^, exhibits nominal expression in leaves under standard nutritional conditions. Contrastingly, its expression is significantly repressed in stems following adjacent foliar fertilization, as shown in Exp. 2, with a nearly 10-fold decrease compared to the control. Similarly, Glyma.13G094900, coding for a xyloglucan endotransglucosylase/hydrolase (XTH), was also notably downregulated in the stems by foliar treatment (Fig. 6). These findings suggest a systemic modulation of the plant’s response to nutritional status mediated through foliar fertilization. The transcriptional repression across both XXT and XTH genes highlights the potential of foliar application in alleviating nutrient deficiency stress.

Spermidine hydroxycinnamoyl transferases (SHTs) are pivotal in various plant functions, such as cell wall modification and specialized metabolism pathways^61,62^. Our research aimed to determine the impact of nutritional deficiency on SHT gene expression. Data from Exps. 1 and 3 revealed that foliar fertilization significantly regulates SHT expression (Glyma.08G312000) in leaves (Suppl. Figs. S2 and S4). These observations suggest that foliar fertilization may counteract the adverse effects of nutritional deficiency by adjusting SHT gene expression to maintain cellular homeostasis.

An uncharacterized gene (Glyma.03G180300) (Suppl. Data S2) was consistently activated by low B treatment, with pronounced suppression after foliar application in all experiments. This gene encodes a small protein containing the LOG motif, which is implicated in converting inactive cytokinins to their active forms^63,64^, suggesting a regulatory role in cytokinin biosynthesis. Cytokinin is known to be influenced by B availability^65^. Interestingly, network analysis in Phytozome v.13 reveals a strong correlation between this gene and a lipid-transfer protein gene (Glyma.18G210900), highlighting a potential interactive pathway in B and cytokinin interplay, particularly within new shoot tissues. Given these associations and the critical involvement of B in cell wall integrity through borate ester cross-linking in RG-II dimers, we propose Glyma.03G180300 as a novel marker for monitoring B nutritional status in soybean, enhancing our understanding of the intricate role of B in plant development and stress response.

### Transcriptional modulation by foliar application of Zn

The expression patterns of genes indicative of low Zn status in soybean leaves subjected to low nutrient conditions showed the influence of foliar-applied Zn on the transcriptional activity. We conducted a comprehensive genomic analysis to identify genes within families known for Zn transport. We also investigated genes responsive to low Zn levels to identify potential biomarkers of Zn status in the plant. While 68 genes were identified in the comparison between low Zn and the complete Hoagland solution in Exp. 1 – Sampling 2, 136 DEGs were found between the low Zn treatment and the foliar application in the same sampling – two of which were identified in both sets (a pectin lyase, Glyma.16G118000, and the C-repeat-binding factor, Glyma.16G199000) as significantly induced by normal levels of Zn or supplementation (Suppl. Data S2 and S5).

#### Potentially relevant membrane transporters regulated by Zn nutritional status

We explored the soybean genome for genes related to Zn or divalent metal transport (Suppl. Data S5 – cf. tab Zn-related genes). We identified 12 genes annotated as ZIP transporters (Zn^2+^-Fe^2+^ Permease family, TCDB #2.A.5), one of which is likely a pseudogene, Glyma.08G063100, which predicted polypeptide of 85 amino acid residues is too short to be a functional membrane transporter and showed null expression across all samples analyzed). A single gene annotated as ZRT/ITR-like was identified in the soybean genome. Three genes annotated as Iron-regulated transporters, one of which is likely a pseudogene (Glyma.16G060200), may also belong to the ZIP family. On the other hand, 142 genes were annotated as Heavy Metal Transport/Detoxification superfamily proteins (ABCB ATPases of the HMT family, TCDB #3.A.1.210). It is possible this list also includes genes coding for other ABCB ATPases associated with divalent metal transport, such as Multidrug Resistance Exporters (MDR, #3.A.1.201).

Vacuolar Iron Transporters (VIT, #2.A.89) have been primarily characterized for their role in transporting iron (Fe) into vacuoles. However, studies have also shown that some members of the VIT family exhibit the ability to also facilitate the transport of other divalent metal ions, including Zn, into vacuoles, such as the Arabidopsis VIT1^27^. While the primary role of VIT transporters is related to Fe, their capability to transport Zn highlights their potential importance in broader micronutrient management strategies in plants. Similarly, natural resistance-associated macrophage proteins (NRAMP) have also been reported to transport Zn^27,66^. We found 12 NRAMP genes in soybean, one of which was not expressed in any of the samples analyzed and another has a very short polypeptide chain allied to very low expression across all samples.

**Fig. 7:**
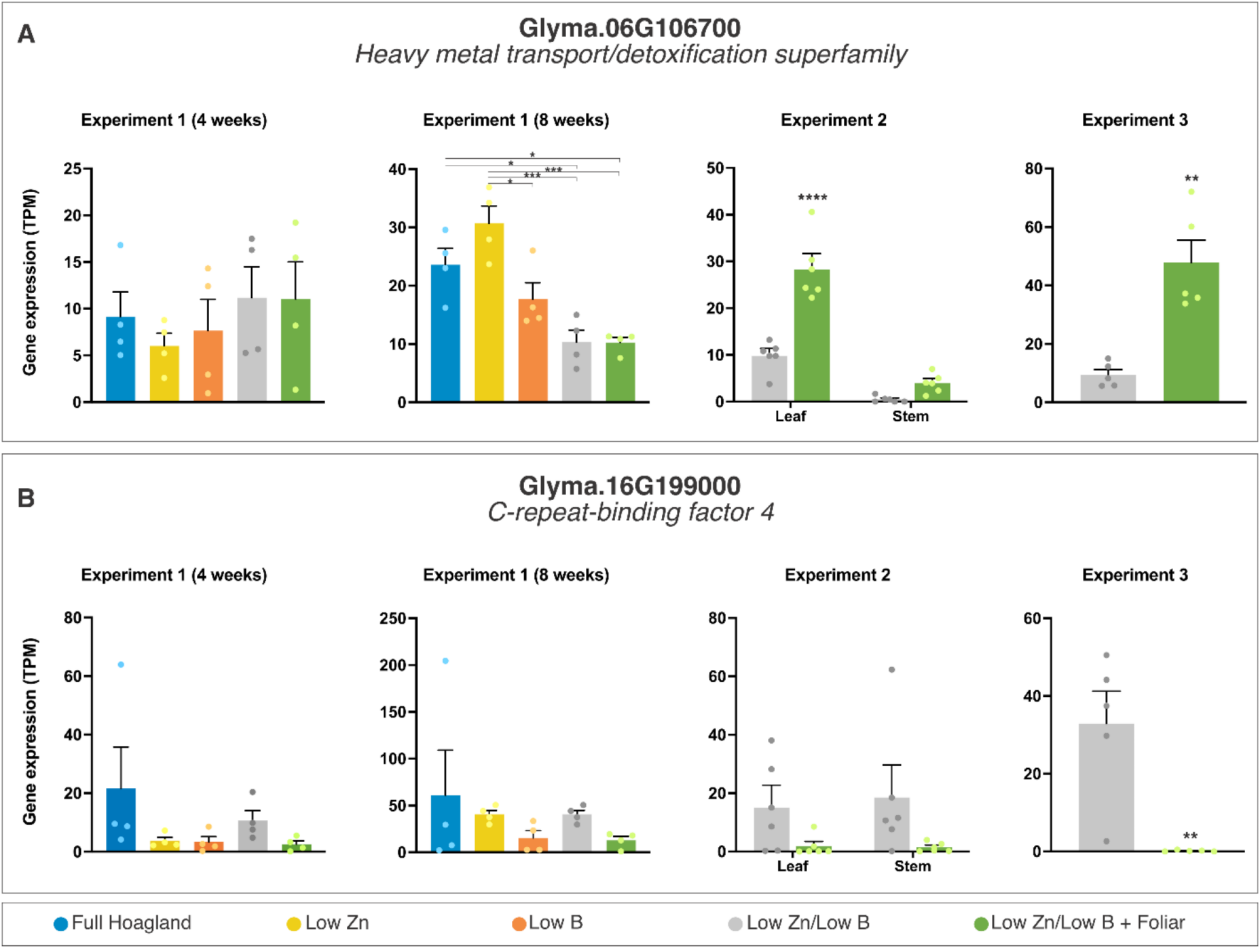
Transcriptional profiles of differently expressed genes (DEGs) regulated by zinc supply across treatments as potential indicators of zinc status in the plant. The data are means ± SEM (*n* ≥ 4 biological replicates) of read counts normalized in transcript per million reads (TPM) for each experimental setup. The asterisks indicate a significant difference between the groups according to Tukey’s test at *p* < 0.05 (*), *p* < 0.01 (**), *p* < 0.001 (***), and *p* < 0.0001 (****).

Among these 193 genes, 23 showed null expression across all samples analyzed. Most of them also display predicted short polypeptide chains (< 150 amino acid residues, except for one HMT and all VIT transporters), most likely indicating pseudogenes. Of the 170 remaining genes with potential function in Zn transport, 6 and 4 appeared as a DEG in Exp. 1 – Sampling 2 when comparing low Zn regimen to the complete Hoagland solution or foliar fertilization, respectively (Suppl. Data S5). Despite its low expression level, a gene coding an HMT transporter (Glyma.06G106700) was consistently induced by low Zn levels compared to the controls (Fig. 7) and has potential as a biomarker indicating Zn deficiency in soybean plants.

## Conclusions

This study indicates that the foliar application of zinc borate as a suspension concentrate fertilizer modulated the gene expression related to B and Zn, suggesting that it can be used as a potential source of foliar micronutrient supply in crop management.

Our findings shed light on how B modulates gene expression of BOR and NIP transporters, as well as cell wall and stress responses in soybean plants. We identified 12 potential BOR and 6 NIP transporter genes, with several displaying differential expressions under modified B availability. The responses involved foliar fertilization under B-deficient conditions using a commercial formulation with B and Zn. Genes encoding cell wall modifying enzymes with known roles in B homeostasis and stress response in model species were significantly modulated by B supplementation via foliar fertilization. Notably, a gene without functional annotation, Glyma.03G180300, emerged as a novel indicator of B status due to its consistent transcriptional response pattern. We also identified genes modulated by Zn supply, including potential transporters.

This study contributes to a better understanding of the complex network of nutritional homeostasis of B and Zn in soybean leaves. It identifies potential genes for future exploration, promising advancements in agricultural sustainability, and the enhancement of crop resilience. Our findings serve as a foundation for future research to dissect the functions of responsive genes across various nutritional and environmental landscapes, ultimately improving micronutrient utilization in soybean plants. This approach underscores the potential for targeted genetic strategies to improve plant nutrition and stress tolerance in crop species.

## Electronic supplementary material

Below is the link to the electronic supplementary material.

Supplementary Material 1

Supplementary Material 2

Supplementary Material 3

Supplementary Material 4

Supplementary Material 5

Supplementary Material 6

Supplementary Material 7

Supplementary Material 8

Supplementary Material 9

Supplementary Material 10

Supplementary Material 11

Supplementary Material 12

Supplementary Material 13

## Data Availability

The complete RNA-Seq dataset generated and analyzed during the current study is available in the NCBI’s SRA repository, BioProject ID PRJNA1164814, and the data is available from the corresponding author upon reasonable request.
