# Supplementary material for: Global transcriptional modulation and nutritional status of soybean plants following foliar application of zinc borate as a suspension concentrate fertilizer
Source: Sci Rep. 2025 Jan 26;15:3309. doi: 10.1038/s41598-025-87771-5 (PMC11770081; doi:10.1038/s41598-025-87771-5)
Supplement: Supplementary file 4 — Supplementary Material 4 [file 41598_2025_87771_MOESM4_ESM.pdf]

## Experiment 1

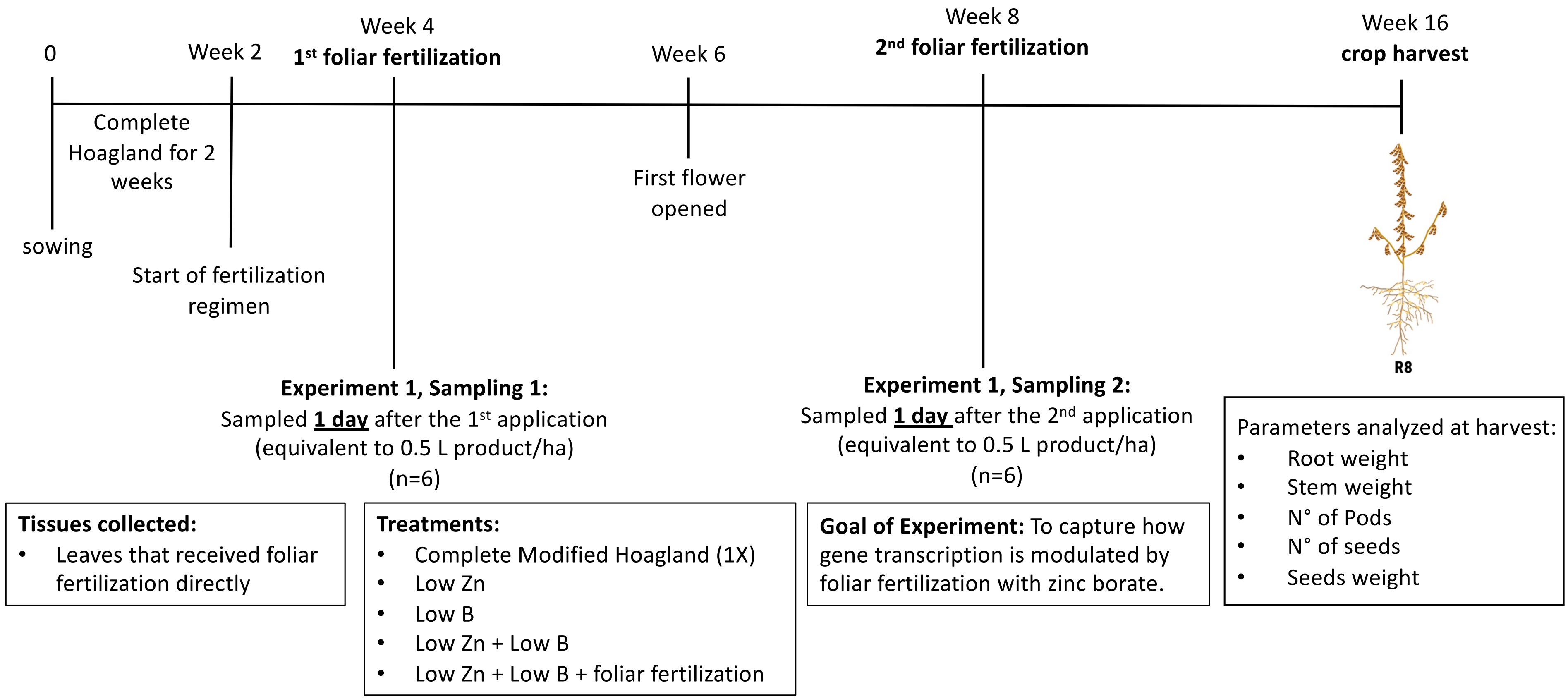


**Suppl. Figure S1.** Setup of Experiment 1: Foliar application of an equivalent of 0.5 L/ha product at 4 and 8 weeks after sowing.
