# Supplementary material for: Global transcriptional modulation and nutritional status of soybean plants following foliar application of zinc borate as a suspension concentrate fertilizer
Source: Sci Rep. 2025 Jan 26;15:3309. doi: 10.1038/s41598-025-87771-5 (PMC11770081; doi:10.1038/s41598-025-87771-5)
Supplement: Supplementary file 5 — Supplementary Material 5 [file 41598_2025_87771_MOESM5_ESM.pdf]

Experiment 3

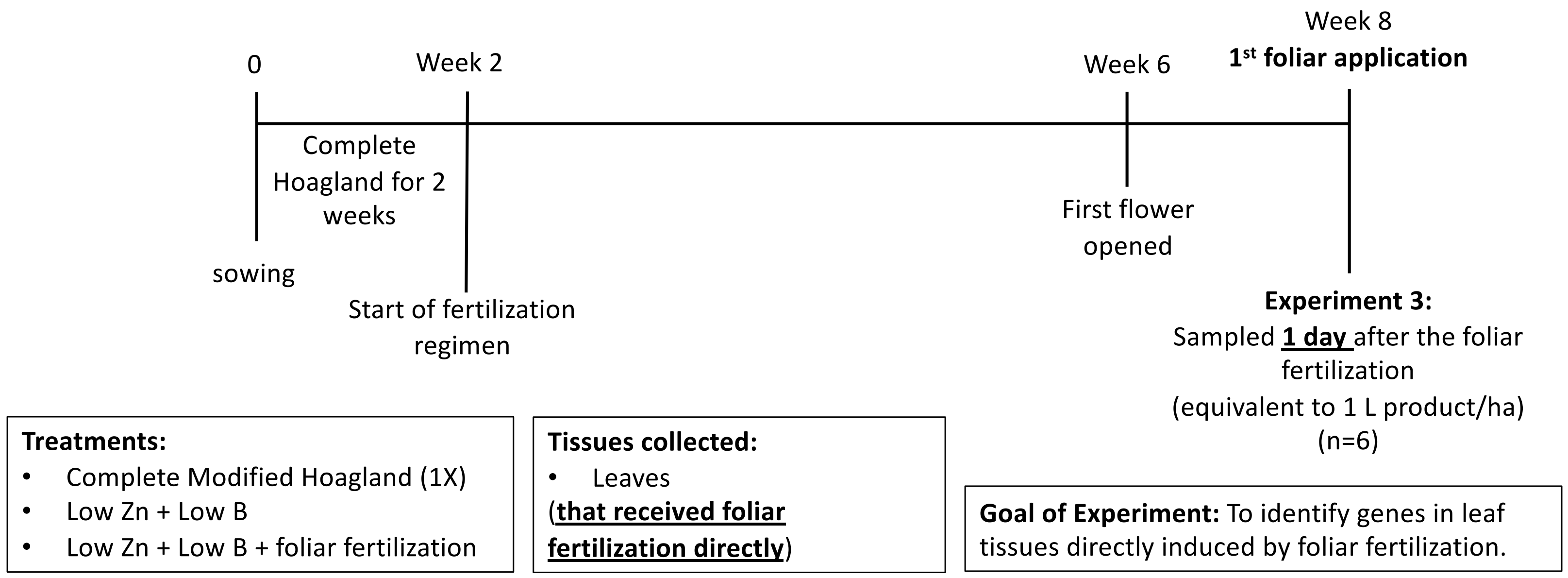

Suppl. Figure S3. Setup of Experiment 3: Foliar application of an equivalent of 1 L/ha product at 8 weeks after sowing. Leaves (n=6) that received foliar fertilization directly were sampled 1 day after foliar application.
