# Supplementary material for: Global transcriptional modulation and nutritional status of soybean plants following foliar application of zinc borate as a suspension concentrate fertilizer
Source: Sci Rep. 2025 Jan 26;15:3309. doi: 10.1038/s41598-025-87771-5 (PMC11770081; doi:10.1038/s41598-025-87771-5)
Supplement: Supplementary file 6 — Supplementary Material 6 [file 41598_2025_87771_MOESM6_ESM.pdf]

Experiment 2

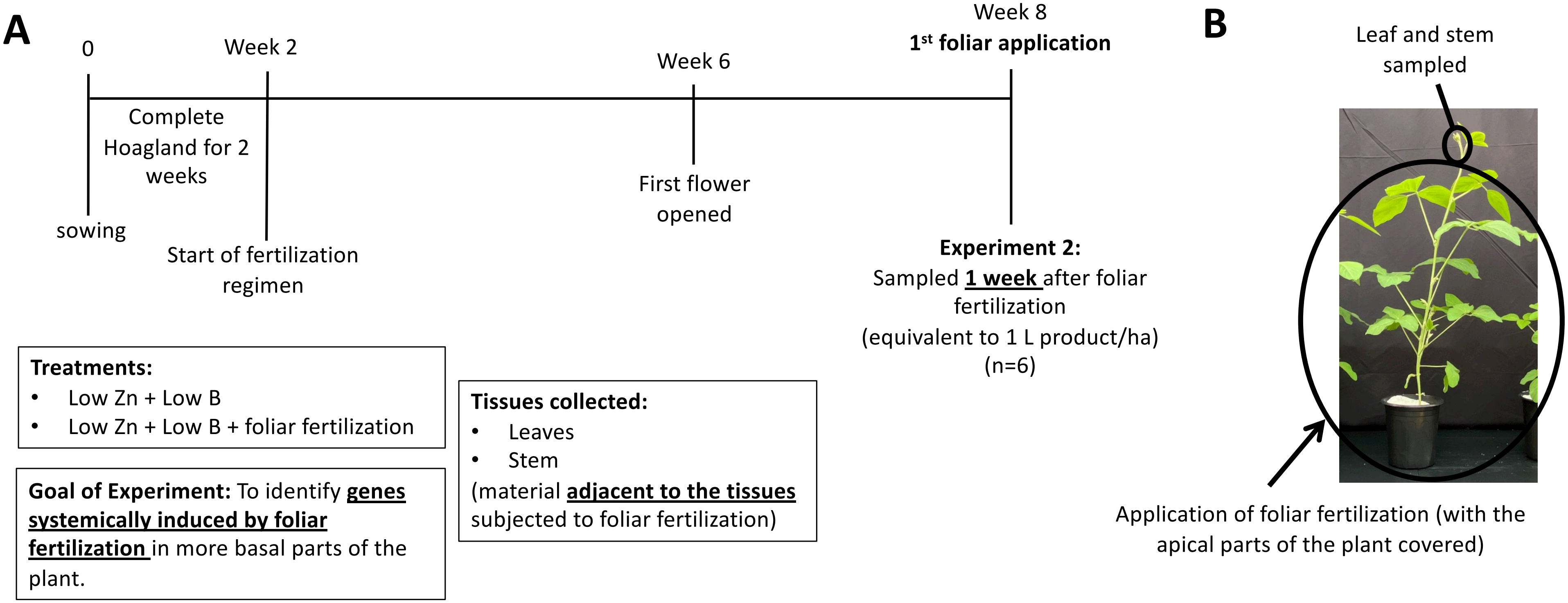

Suppl. Figure S2. (A) Setup of Experiment 2: Foliar application of an equivalent of 1 L/ha product at 8 weeks after sowing. Leaves and stems (n=6) above the applied area were sampled 1 week after foliar application. **(B)** Visual scheme of application of foliar fertilization and tissue sampling.
