# Supplementary material for: Global transcriptional modulation and nutritional status of soybean plants following foliar application of zinc borate as a suspension concentrate fertilizer
Source: Sci Rep. 2025 Jan 26;15:3309. doi: 10.1038/s41598-025-87771-5 (PMC11770081; doi:10.1038/s41598-025-87771-5)
Supplement: Supplementary file 7 — Supplementary Material 7 [file 41598_2025_87771_MOESM7_ESM.pdf]

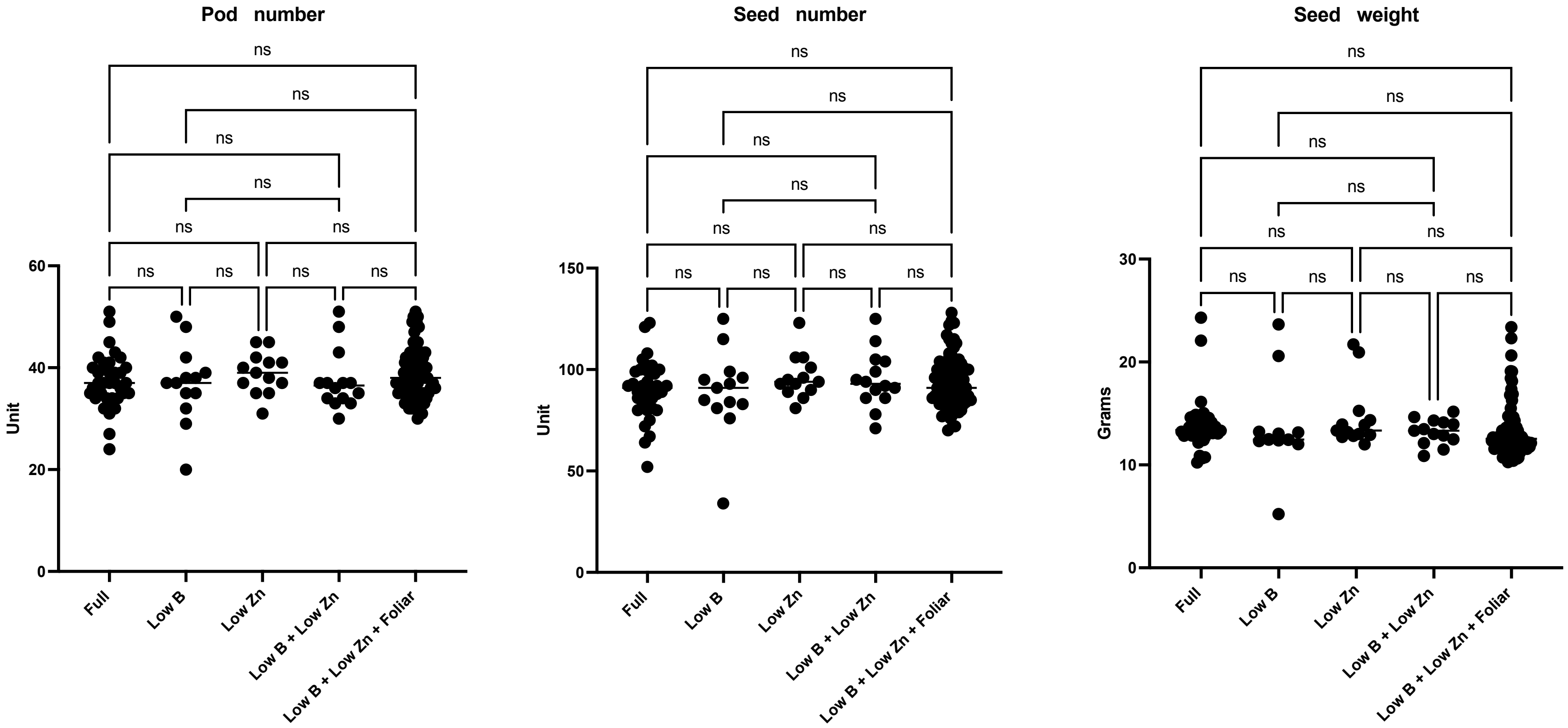

Suppl. Figure S5. Agronomic parameters per plant at harvest of soybean plants at week 16. No statistically significant differences were observed among the treatments with the Tukey's test at 95% confidence.
