# Supplementary material for: Global transcriptional modulation and nutritional status of soybean plants following foliar application of zinc borate as a suspension concentrate fertilizer
Source: Sci Rep. 2025 Jan 26;15:3309. doi: 10.1038/s41598-025-87771-5 (PMC11770081; doi:10.1038/s41598-025-87771-5)
Supplement: Supplementary file 9 — Supplementary Material 9 [file 41598_2025_87771_MOESM9_ESM.pdf]

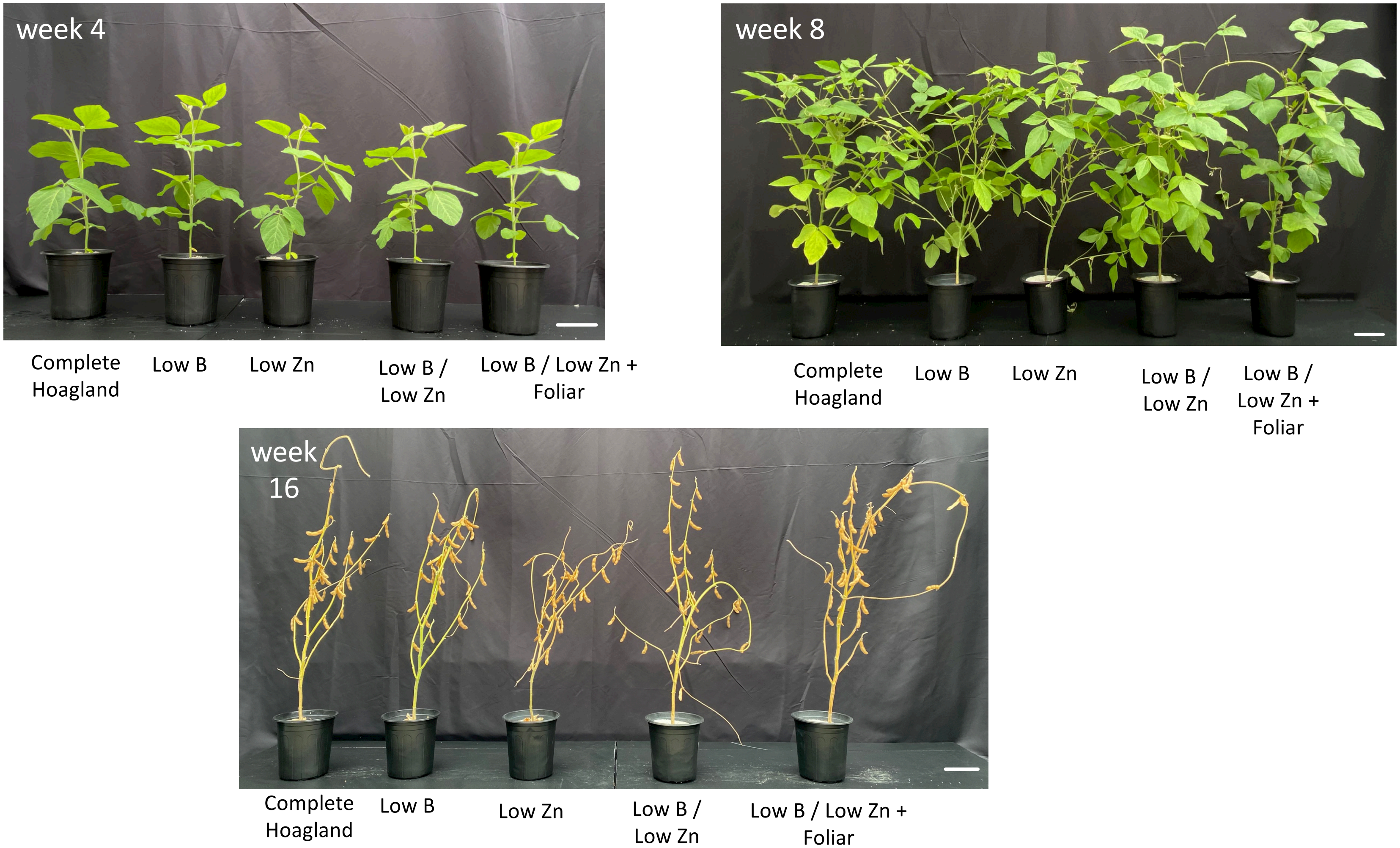

Suppl. Figure S4. Development of representative plants under each treatment during the soybean cycle. Scale: 10 cm
