# Supplementary material for: Global transcriptional modulation and nutritional status of soybean plants following foliar application of zinc borate as a suspension concentrate fertilizer
Source: Sci Rep. 2025 Jan 26;15:3309. doi: 10.1038/s41598-025-87771-5 (PMC11770081; doi:10.1038/s41598-025-87771-5)
Supplement: Supplementary file 10 — Supplementary Material 10 [file 41598_2025_87771_MOESM10_ESM.pdf]

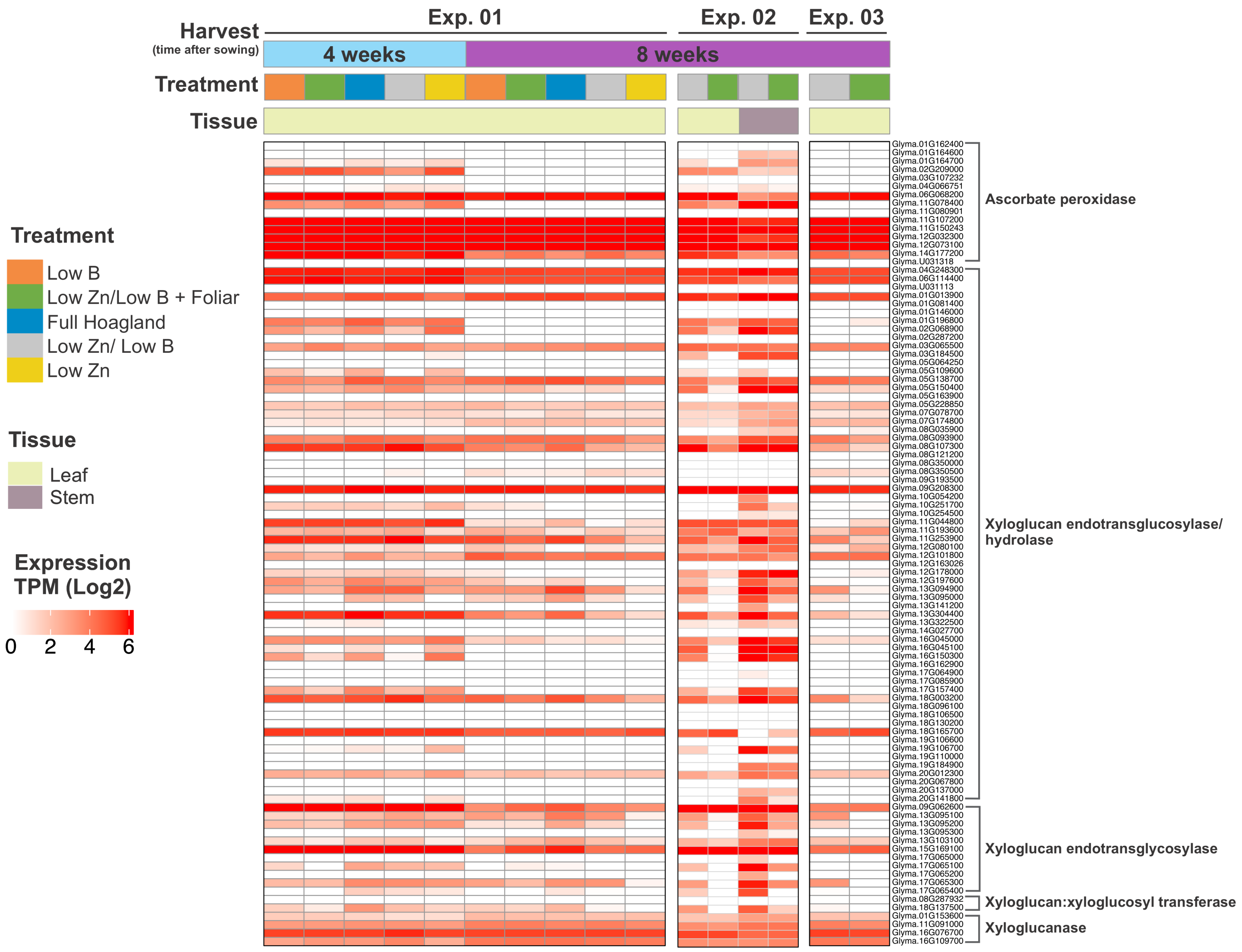

Supplemental Figure S7. Heatmap of normalized gene expression mean (log-transformed transcripts per million) for members of gene families involved in boron physiology.
